# Immunosenescence modulates radiation-induced anti-tumor immunity: implications for personalized immuno-oncology

**DOI:** 10.3389/fimmu.2026.1888282

**Published:** 2026-07-10

**Authors:** Christoph R. Arnold, Julian Mangesius, Birgit Weinberger, Iana Portnaia, Antonio Santacroce, Ute Ganswindt, Alexander Muacevic

**Affiliations:** 1 European Radiosurgery Center Munich, Munich, Germany; 2 Department of Radiation Oncology, Medical University of Innsbruck, Innsbruck, Austria; 3 Research Institute for Biomedical Aging Research, Universität Innsbruck, Innsbruck, Austria; 4 Department of Internal Medicine II, Medical University of Innsbruck, Innsbruck, Austria; 5 Institute of Human Genetics, Medical University of Innsbruck, Innsbruck, Austria; 6 Department of Neurosurgery, St. Barbara-Klinik Hamm-Heessen, Hamm, Germany; 7 Department of Medicine, Faculty of Health, Witten/Herdecke University, Witten, Germany

**Keywords:** abscopal effect, anti-tumor immunity, immune checkpoint inhibitors, immuno senescence, personalized immuno-oncology, radiation oncology, radio-immunotherapy, tumor-microenvironment

## Abstract

Radiotherapy (RT) has evolved from a purely cytotoxic treatment into a potent immune modulator capable of inducing systemic anti-tumor responses. High-dose precision approaches such as stereotactic body radiotherapy or stereotactic radiosurgery can trigger immunogenic cell death, enhance antigen presentation, and promote tumor-specific T-cell priming. However, the magnitude and quality of these radiation-induced immune effects depend critically on the biological age of the host immune system. Immunosenescence—defined by the gradual decline and remodeling of immune competence with age—profoundly alters both innate and adaptive immunity. The aged immune landscape is characterized by reduced responsiveness, chronic low-grade inflammation, and impaired coordination between immune effector cells. In this context, RT may not elicit the same immunogenic signals observed in younger patients. Instead, aging-associated immune alterations can dampen the anti-tumor potential of radiation or shift the balance toward prolonged inflammation and immune dysregulation. These effects highlight the need to consider immune aging as a key determinant of therapeutic response. Understanding how immunosenescence modulates radiation-induced immunity is essential for developing age-informed and immune-adaptive RT strategies. Integrating biomarkers of immune aging into treatment planning could enable truly personalized immuno-oncology approaches—optimizing efficacy, minimizing toxicity, and improving outcomes in older patients with cancer.

## The anti-tumor immune response in context of radiotherapy

### Core mechanistic pathways of radiation-induced anti-tumor immunity

**Figure 1:**
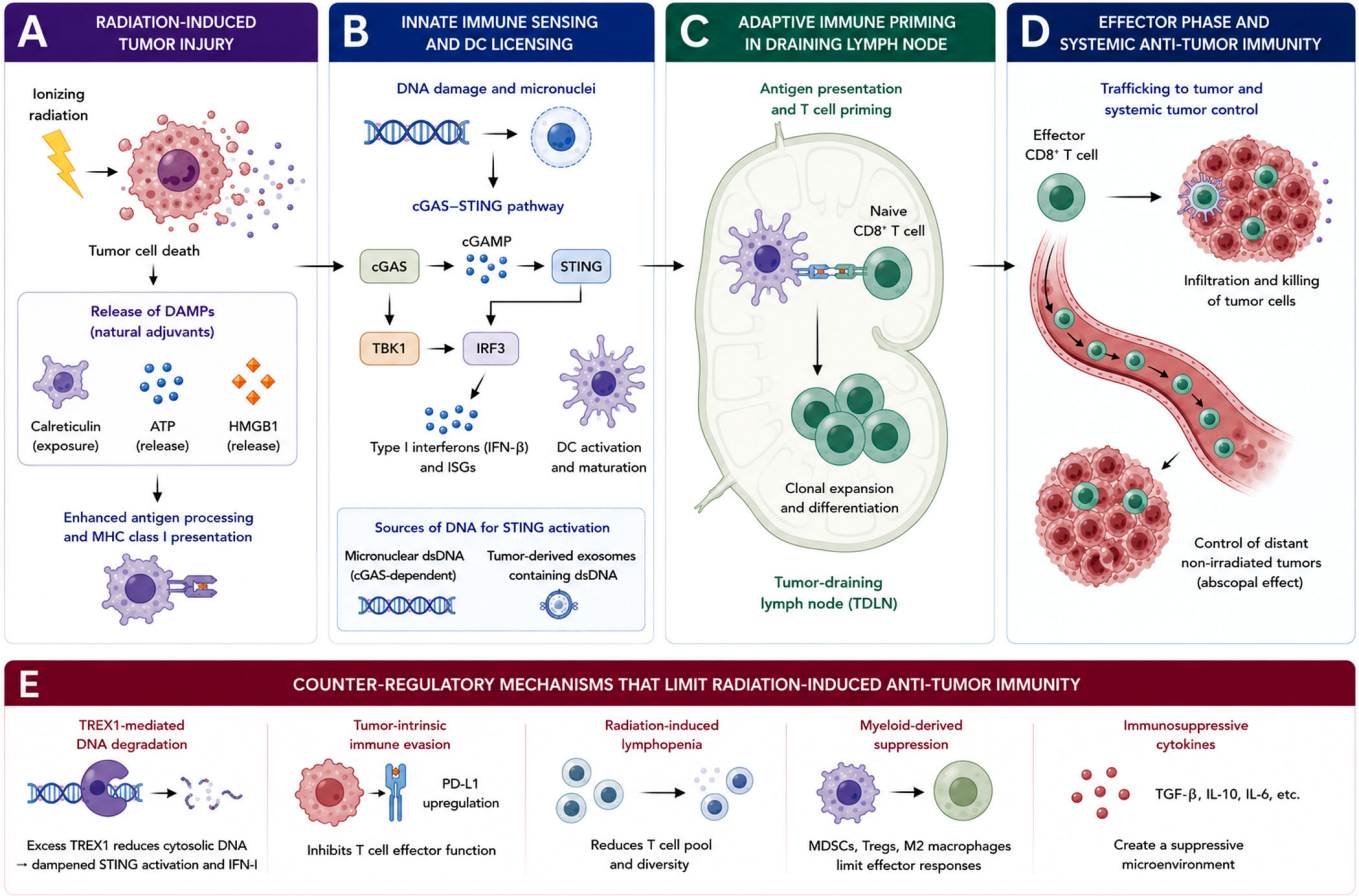
Radiotherapy as an *in situ* vaccine: from local tumor injury to systemic anti-tumor immunity. **(A)** Ionizing radiation induces tumor cell death and promotes immunogenic cell death (ICD), leading to the release and exposure of damage-associated molecular patterns (DAMPs), including calreticulin, ATP, and High Mobility Group Box 1 (HMGB1). These signals enhance antigen processing and MHC class I presentation. **(B)** Radiation-induced DNA damage and micronuclei formation activate innate immune sensing pathways through cGAS-STING signaling, resulting in type I interferon production, dendritic cell (DC) activation, and DC maturation. Cytosolic DNA may originate from micronuclear rupture or tumor-derived exosomes containing double-stranded DNA (dsDNA). **(C)** Activated DC mediate antigen presentation and cross-priming of naïve CD8+ T cells within the tumor-draining lymph node (TDLN), leading to clonal expansion and differentiation of tumor-specific effector T cells. **(D)** Effector CD8+ T cells traffic back to the tumor site, mediate tumor cell killing, and may contribute to systemic anti-tumor responses, including control of distant non-irradiated lesions (abscopal effects). **(E)** Multiple counter-regulatory mechanisms can limit radiation-induced anti-tumor immunity, including Three-Prime Repair Exonuclease 1 (TREX1)-mediated degradation of cytosolic DNA, tumor-intrinsic immune evasion through Programmed Death-Ligand 1 (PD-L1) upregulation, radiation-induced lymphopenia, myeloid-derived suppressive mechanisms, and immunosuppressive cytokines such as TGF-β, IL-10, and IL-6. Together, these pathways may dampen STING activation, impair effector T-cell function, and promote an immunosuppressive tumor microenvironment.

Radiotherapy (RT) can promote anti-tumor immunity not only through direct clonogenic tumor cell kill, but also by converting the irradiated lesion into an *in-situ* vaccine, in which tumor destruction provides both antigenic material and endogenous adjuvant signals for adaptive immune priming (1). This cascade links immunogenic tumor injury, innate sensing, dendritic-cell (DC) activation, and cross-priming of tumor-reactive CD8+ T cells, thereby supporting local and, under favorable conditions, systemic tumor control (1, 2). The major mechanistic steps and counter-regulatory pathways underlying RT-induced anti-tumor immunity are illustrated in **Figure 1**.

A first essential step is immunogenic tumor injury. Irradiated tumor cells expose or release damage-associated molecular patterns (DAMPs), including calreticulin, ATP, and High Mobility Group Box 1 (HMGB1), which promote phagocytic uptake and inflammatory antigen presentation (2, 3). In parallel, RT enhances antigen processing and MHC class I peptide presentation, increasing the visibility of surviving tumor cells to cytotoxic T lymphocytes (2, 4). Thus, RT supplies both tumor antigens and the inflammatory context required for productive anti-tumor immunity.

The dominant innate pathway connecting radiation-induced genomic injury to adaptive immunity is the cGAS-STING-type I interferon axis. Ionizing radiation induces DNA damage, mitotic errors, and micronuclei; upon micronuclear rupture, cytosolic double-stranded DNA is sensed by cGAS, leading to cGAMP production, STING-TBK1-IRF3 activation, and induction of IFN-β and interferon-stimulated genes (1, 5, 6). Deng et al. showed that host STING is required for radiation-induced type I interferon production and anti-tumor efficacy and identified CD11c+ dendritic cells as major IFN-β producers after RT (1).

This pathway also links RT-induced DNA damage to cellular senescence. cGAS-dependent sensing of damaged or displaced chromatin can promote senescence-associated inflammatory signaling, whereas Three-Prime Repair Exonuclease 1 (TREX1) deficiency enhances cytosolic DNA accumulation, cGAS–STING activation, and irradiation-induced senescence in experimental models. However, these findings derive primarily from cellular and genetic-deficiency models, and direct evidence that physiological aging alters RT-induced TREX1 expression or the radiation-dose threshold at which TREX1 suppresses IFN-I signaling in tumors remains lacking (7–9).Type I interferon signaling is functionally critical because it licenses DCs for cross-priming. DCs lacking cGAS-STING signaling show impaired sensing of irradiated tumor cells and reduced priming of tumor-specific CD8+ T cells, whereas exogenous IFN-β restores this capacity (1). This is particularly relevant for cDC1 populations, which specialize in MHC class I cross-presentation and are central to priming naïve CD8+ T cells against tumor antigens (1, 10–12). Productive anti-tumor priming also depends on conventional CD4+ helper T cells, which license antigen-presenting cells through CD40–CD40L interactions and provide cytokine support for CD8+ T-cell expansion, effector differentiation, and memory formation. Age-associated DC dysfunction, contraction of the naïve CD4+ T-cell pool, and impaired helper-cell activation may therefore weaken the conversion of RT-induced innate sensing into durable adaptive immunity (13–15). This connection remains incompletely characterized in RT-specific aging models. RT-derived nucleic acid signals can also reach antigen-presenting cells through extracellular vesicles; exosomes from irradiated tumor cells contain double-stranded DNA that activates STING-dependent signaling in DCs and promotes tumor-specific CD8+ T-cell responses (16).

The tumor-draining lymph node (TDLN) is the key anatomical site where local radiation-induced injury is translated into systemic adaptive immunity. After antigen uptake and IFN-I-driven maturation, DCs migrate to the TDLN, cross-present tumor-derived peptides, and initiate clonal expansion of tumor-reactive CD8+ T cells. Preservation of the TDLN supports systemic tumor control, whereas disruption of nodal priming impairs this process (1, 10, 17).

Once primed, tumor-reactive CD8+ T cells expand and traffic back to the irradiated lesion and, under favorable conditions, to distant non-irradiated tumor sites. This effector phase provides the mechanistic basis for systemic tumor control and, in rare cases, the abscopal effect (1, 10). Overall, RT acts as an *in-situ* vaccine only when immunogenic tumor injury, cGAS-STING-dependent innate licensing, DC-mediated cross-priming, and T-cell trafficking remain coupled; failure at any step can abort the transition from local radiation injury to systemic anti-tumor immunity. A comprehensive overview of the distinct anatomical compartments and immunologic steps involved in RT-induced anti-tumor immunity is summarized in **Table 1**.

**Table 1:** Spatially organized map of radiotherapy-induced anti-tumor immunity.

Compartment	Key RT-induced events	Main cells involved	Dominant mediators/pathways	Functional output	Failure point if impaired
Irradiated tumor (2–4)	DNA damage, tumor stress, immunogenic cell death, antigen release, calreticulin exposure, ATP/HMGB1 release	Tumor cells	DNA double-strand breaks, ER stress, DAMP release	Generates antigenic substrate and danger signals for immune recognition	Poor immunogenic cell death; weak antigen release
Tumor microenvironment (1, 5)	Cytosolic dsDNA accumulation, micronuclei formation, innate sensing after RT	Tumor cells, stromal cells, host APCs	cGAS-STING, TBK1-IRF3, type I IFN induction	Converts local radiation injury into an innate inflammatory/adjuvant signal	TREX1 induction after excessive single-fraction dose; suppressive myeloid remodeling
Dendritic-cell compartment (1, 16, 18)	Antigen uptake, sensing of irradiated tumor-derived material, maturation, migration	cDC1/Batf3-dependent DCs, CD11c+ DCs	IFN-β, costimulatory molecules, STING-dependent licensing, exosomal dsDNA transfer	Enables efficient cross-presentation of tumor antigens	DC dysfunction, impaired migration, defective STING/IFN-I signaling
Tumor-draining lymph node (TDLN) (1, 10, 19)	Cross-priming of naïve T cells, early clonal expansion, maintenance of stem-like tumor-reactive T cells	DCs, naïve CD8+ T cells, early activated T cells	MHC-I cross-presentation, TCR signaling, IFN-I-supported priming	Generates tumor-specific effector CD8+ T-cell pool and seeds systemic immunity	Elective nodal irradiation, reduced naïve T-cell pool, impaired priming niche
Peripheral blood/systemic immune compartment (23, 24)	Expansion, circulation, and maintenance of tumor-reactive lymphocytes	Activated CD8+ T cells, memory precursors, circulating lymphocytes	Cytokine support, trafficking programs, systemic immune surveillance	Disseminates anti-tumor immunity beyond the radiation field	Radiation-induced lymphopenia, repertoire restriction, immune exhaustion
Distant non-irradiated tumor (20, 22, 38)	Effector T-cell infiltration, recognition of shared tumor antigens, cytotoxic killing	CD8+ T cells, tumor cells, local myeloid and stromal cells	TCR-mediated recognition, perforin/granzyme pathways, IFN-γ	Basis of systemic tumor control and the abscopal effect	Poor trafficking, PD-L1 induction, suppressive TME, myeloid barriers

Rather than occurring as a purely linear sequence, radiotherapy (RT)-induced anti-tumor immunity unfolds across distinct anatomical compartments. In the irradiated tumor, radiation generates tumor antigens and damage associated molecular patterns (DAMPs). Within the tumor microenvironment (TME), cytosolic DNA sensing through the cGAS-STING pathway induces type I interferon programs. These signals license dendritic cells for antigen uptake, maturation, and migration to the tumor-draining lymph node (TDLN), where cross-priming and early expansion of tumor-reactive CD8+ T cells occur. Activated lymphocytes then circulate systemically and may infiltrate distant, non-irradiated lesions, mediating the abscopal effect. Each compartment contains distinct failure points, including Three-Prime Repair Exonuclease 1 (TREX1) induction, dendritic-cell dysfunction, TDLN damage, lymphopenia, immunosenescence, and suppressive remodeling of the TME.

### Dose/fractionation and radiobiological determinants of radiation-induced anti-tumor immunity

Dose and fractionation are immunologic determinants rather than neutral technical variables. In the context of RT as an *in-situ* vaccine, the selected regimen influences whether irradiation sustains tumor injury, innate sensing, dendritic-cell recruitment, and T-cell priming, or whether this cascade is curtailed by radiobiological counter-regulation (5, 18–20). Thus, the relevant issue is not only tumor cell kill, but whether the schedule preserves the conditions required for immunogenicity.

A central determinant is the persistence of cytosolic double-stranded DNA after irradiation. Vanpouille-Box et al. showed that high single fractions induce the exonuclease TREX1, which degrades radiation-generated cytosolic dsDNA, suppresses cGAS-STING activation, and reduces IFN-β production (5). TREX1 induction generally occurred at approximately 12–18 Gy per fraction, whereas repeated doses below this threshold, exemplified by 8 Gy × 3, sustained cytosolic DNA, type I interferon programs, and Batf3-dependent dendritic-cell recruitment (5). This provides a molecular explanation for why larger single fractions are not necessarily more immunogenic than moderate hypofractionation.

Experimental senescence models suggest that impaired TREX1-dependent DNA clearance can amplify the inflammatory consequences of radiation-induced DNA damage. Reduced TREX1 activity promotes cytosolic DNA accumulation, cGAS–STING signaling, and (senescence-associated secretory phenotype) SASP expression, while TREX1 deficiency enhances irradiation-induced DNA damage and senescence in murine fibroblasts. However, these findings do not demonstrate that physiological aging changes the approximately 12–18 Gy threshold for RT-induced TREX1 expression. Age-specific fractionation recommendations therefore cannot currently be derived from TREX1 biology alone (9, 21).

Preclinical combination studies support the importance of fractionation. Dewan et al. showed that fractionated RT, but not a biologically comparable single dose, induced an immune-mediated abscopal effect when combined with anti-CTLA-4; in that model, 8 Gy × 3 outperformed 6 Gy × 5, whereas 20 Gy × 1 failed to generate the same systemic response (20). Similarly, Lee et al. showed that ablative RT increased T-cell priming in draining lymphoid tissues and that local tumor control depended on CD8+ T cells, whereas conventional fractionation and adjuvant chemotherapy diminished these immune effects (19). Gupta et al. further demonstrated that a single 10 Gy fraction could mobilize tumor-specific immunity, but only when DCs and CD8+ T cells were intact (18).

Together, these studies suggest that immunogenic fractionation operates within a therapeutic window: the dose must be sufficient to induce immunogenic tumor stress and antigen presentation, but not so high that TREX1 extinguishes upstream DNA sensing. Fractionation also shapes adaptive resistance, as Dovedi et al. showed that low-dose fractionated RT upregulated Programmed Death-Ligand 1(PD-L1) and created an acquired resistance program that was overcome by concurrent, but not delayed, PD-L1 blockade (22). Thus, the same schedules that promote priming may also induce checkpoint-mediated suppression.

Overall, the most immunogenic regimens appear to generate sufficient tumor stress, preserve cytosolic DNA-driven IFN-I signaling below the TREX1 threshold, and allow DC recruitment and T-cell priming before suppressive feedback or lymphocyte depletion dominates (5, 18–20, 22). In many preclinical systems, moderate hypofractionation, particularly 8 Gy × 3, best fits this model (5, 20). The magnitude of the resulting immune response may nevertheless depend on the host’s capacity to translate IFN-I signaling into effective dendritic-cell and T-cell responses, although direct age-stratified comparisons remain lacking. Dose and fractionation should therefore be selected according to both tumor radiobiology and the intended immune program, while recognizing that current evidence does not support a distinct age-specific fractionation regimen. The immunologic consequences of different RT schedules are summarized in **Table 2**.

**Table 2:** Dose and fractionation determinants of radiotherapy-induced anti-tumor immunity.

Fractionation strategy	Typical example	TREX1 induction	Cytosolic dsDNA persistence	Type I IFN induction	DC activation	Cross-priming potential	Lymphopenia risk	Immunosuppressive feedback	Overall immunogenic potential
High single fraction	20 Gy × 1	↑	↓	↓	±	±/↓	↓	±	Intermediate
Moderate hypofractionation	8 Gy × 3	↓	↑	↑	↑	↑	±	±/↑	High
Conventional fractionation	2 Gy × 25–30	↓	±/↓	±/↓	±	±/↓	↑	↑	Low–intermediate

Qualitative scoring is based on primary preclinical evidence showing that high single fractions above the Three-Prime Repair Exonuclease 1 (TREX1)-inducing threshold reduce cytosolic dsDNA and IFN-I signaling, whereas moderate hypofractionation (exemplified by 8 Gy × 3) better sustains dendritic-cell recruitment and cross-priming. Conventional fractionation is immunologically heterogeneous and may be limited by cumulative lymphopenia and adaptive suppressive feedback.

### Immunosuppressive effects and counter-regulation

RT-induced immunity is bidirectional: the same treatment that initiates vaccine-like anti-tumor responses can also activate compensatory programs that limit their amplitude, durability, and systemic spread. These include checkpoint induction, expansion of regulatory T cells (Tregs), suppressive myeloid remodeling, treatment-related lymphocyte depletion, and host factors such as immunosenescence (13, 14, 22–25).

A major suppressive pathway is PD-L1 upregulation. Dovedi et al. showed that low-dose fractionated RT increased PD-L1 expression across several syngeneic models and created an acquired resistance state; concurrent, but not delayed, PD-L1 blockade restored CD8+ T-cell-dependent tumor control, improved survival, and generated immunologic memory (22). Thus, RT-induced checkpoint expression represents a mechanistically relevant feedback loop rather than a secondary epiphenomenon.

PD-L1 induction occurs within broader suppressive remodeling of the tumor microenvironment (TME). In a non-small cell lung cancer model, Gong et al. showed that anti-PD-L1 plus RT improved tumor control compared with either treatment alone and was associated with increased CD8+ T-cell infiltration and fewer myeloid-derived suppressor cells (MDSCs) (25). These findings suggest that checkpoint blockade can relieve T-cell inhibition while also reshaping myeloid barriers that constrain RT-induced immunity.

Tregs represent an additional mechanism of radiobiological counter-regulation. Because Tregs may be relatively radioresistant compared with conventional lymphocytes, RT can increase their proportional representation within the remaining T-cell compartment. Kachikwu et al. observed Treg enrichment after irradiation, while Muroyama et al. demonstrated an increase in functionally suppressive intratumoral Tregs after stereotactic RT (26, 27). Beyond differential radiosensitivity, chemokine-dependent recruitment may sustain Treg accumulation within tumors. In primary breast cancer, Gobert et al. identified selective CCL22/CCR4-mediated recruitment and local activation of suppressive Tregs in peritumoral lymphoid infiltrates, which was associated with adverse clinical outcomes (28). Zhu et al. further summarized the context-dependent effects of RT-induced TME remodeling on Tregs and other suppressive immune populations (29). Tregs also accumulate in several aged-host settings, although age-associated changes in their phenotype and suppressive function are tissue- and context-dependent (30, 31). An increased Treg-to-effector T-cell ratio could therefore further restrict RT-induced priming and cytotoxic activity in older adults.

Another major barrier is radiation-induced lymphopenia (RIL). Because lymphocytes are highly radiosensitive and continuously recirculate through blood, marrow, lymphoid organs, and irradiated tissues, RT can deplete cells required for antigen-specific tumor control. In patients with non-small cell lung cancer, Tang et al. linked severe treatment-related lymphopenia to larger tumor volume, greater low-dose lung exposure, and inferior survival (23). RIL may impair T-cell precursor availability, clonal expansion after cross-priming, and systemic immune surveillance. This may be particularly consequential in older adults, because age-associated contraction of the naïve CD4+ and CD8+ T-cell pools, reduced proliferative capacity, and impaired metabolic activation may limit recovery after treatment-related lymphocyte depletion (30, 32–35).

Overall, the limited systemic efficacy of RT alone reflects interacting suppressive forces, including PD-L1-mediated adaptive resistance, Treg enrichment, suppressive myeloid remodeling, lymphocyte depletion, and age-associated impairment of immune regeneration and effector function (13, 14, 22–25). These barriers do not negate the *in-situ* vaccine concept but explain why RT-induced immune activation often requires therapeutic support to become durable and systemic (13, 14, 22–25).

### Kinetics and timing of the radiation-induced immune cascade

RT-induced immunity unfolds as a temporally ordered cascade rather than a single event. Within minutes to the first 1–3 days, irradiation induces tumor stress, DNA damage signaling, DAMP exposure or release, and immunogenic modulation, including calreticulin, ATP, HMGB1, enhanced antigen processing, and increased MHC class I presentation (2–4). Gameiro et al. detected these immunogenic changes 72 hours after irradiation (2).

This early phase overlaps with innate nucleic acid sensing and type I interferon induction. Deng et al. showed that host STING is required for RT-induced IFN-β production and immune priming, with intratumoral IFN-β detectable by approximately day 3 (1). Jagodinsky et al. further showed that IFN-I activation is model-dependent, peaking at day 1 in MOC2 tumors and around day 7 in B16 and B78 melanoma models (36).

Over the next several days, DCs acquire antigen, mature, and migrate to the TDLN, consistent with broader DC migration kinetics of approximately 1–3 days (37). There, DCs cross-present tumor antigens and prime CD8+ T cells. In Deng et al., antigen-specific CD8+ T-cell responses were assessed around day 8 after RT, while Buchwald et al. showed that preservation of the TDLN is essential for robust abscopal responses and maintenance of stem-like tumor-specific CD8+ T cells (1, 10). In older hosts, impaired DC function and contraction of the naïve T-cell pool may attenuate the transition from innate sensing to adaptive priming (13, 14, 24). Whether immune aging also alters the timing of this transition has not been established, because direct comparisons of RT-induced immune kinetics in young and older hosts remain scarce.

The effector phase then develops over days to weeks, as tumor-reactive CD8+ T cells traffic to irradiated and distant tumor sites, enabling tumor control beyond direct cytotoxicity and, occasionally, abscopal responses (10, 18). However, suppressive feedback emerges in parallel: fractionated RT induces PD-L1 upregulation and adaptive resistance, making concurrent checkpoint blockade more effective than delayed treatment (22).

Thus, RT-induced immunity proceeds through overlapping waves of tumor injury, IFN-I induction, DC migration and T-cell priming, followed by effector trafficking and adaptive resistance. These intervals are approximate and model-dependent, and host immune age may modify the magnitude and potentially the kinetics of the response. This should be considered when scheduling combination treatments: innate- or DC-directed interventions may be most relevant early, whereas checkpoint blockade should overlap with the priming and effector phases during which adaptive resistance emerges (1, 10, 22, 36). Key temporal phases of the RT-induced immune response are outlined in **Table 3**.

**Table 3:** Kinetic timeline of the radiotherapy-induced immune cascade.

Time after RT	Dominant events	Main compartment	Key readouts/biomarkers	Therapeutic relevance
Minutes to hours	DNA damage response, ER stress, onset of tumor stress signaling	Irradiated tumor cells	DNA damage markers, stress signaling	Sets stage for immunogenic injury
­­~0–3 days	DAMP exposure/release; calreticulin, ATP, HMGB1; early innate activation	Tumor bed	Calreticulin exposure, ATP/HMGB1 release	Window for maximizing immunogenic tumor injury
~Days 1-7	IFN-I induction through cGAS-STING	Tumor and host APC compartments	IFN-β, ISGs, STING/TBK1/IRF3 activation	Critical window for innate adjuvant programming
~Days 2–5	DC activation and migration from tumor to TDLN	cDC1/DC compartment, lymphatics, TDLN	Costimulatory markers, Batf3-dependent DC accumulation	Supports sequencing of agents aimed at boosting DC function
~Days 5–8	Cross-priming of tumor-specific CD8+ T cells	Tumor-draining lymph node	Antigen-specific CD8 priming assays, T-cell activation markers	Central step for generation of systemic immunity
~1–3 weeks	Clonal expansion, trafficking to tumor, effector tumor killing	Systemic T-cell pool, tumor sites	Increased CD8 infiltration, tumor regression, distant lesion responses	Window in which abscopal responses may become detectable
days to weeks	Emergence of adaptive resistance	Tumor microenvironment	PD-L1 upregulation, suppressive myeloid signals	Supports concurrent or closely sequenced checkpoint blockade
Late phase	Memory formation or immune failure	Memory T-cell pool; residual tumor	Durable control vs relapse	Defines long-term success of *in situ* vaccination

Representative, model-dependent temporal framework of radiation-induced anti-tumor immunity. Following irradiation, tumor stress and immunogenic cell death-associated signals emerge within minutes to days, including damage-associated molecular pattern (DAMP) exposure and release. This is followed by cGAS-STING-dependent type I interferon induction, dendritic-cell activation and migration to the tumor-draining lymph node (TDLN), cross-priming of tumor-specific CD8+ T cells, and subsequent effector expansion and trafficking to irradiated and distant tumor sites. Overlapping with these immunostimulatory phases, adaptive suppressive programs such as Programmed Death-Ligand 1 (PD-L1) upregulation may also emerge. The indicated time windows are approximate and derived from representative preclinical studies; exact kinetics vary by tumor model, host immune state, dose, and fractionation.

### Systemic immunity, the abscopal effect, and combinations of radiotherapy and immune checkpoint inhibitors

The systemic manifestation of RT-induced immunity is the abscopal effect, defined as regression of non-irradiated lesions after localized RT (38). Although biologically compelling, it remains rare with RT alone, suggesting that antigen release and innate sensing are usually insufficient without preserved tumor-draining lymph node function, adequate systemic T-cell reserves, and relief of checkpoint- or myeloid-mediated suppression (10, 20, 22, 23, 38). Age-associated contraction of the naïve T-cell pool, impaired DC function, and restricted immune repertoire may further reduce the probability that local RT generates a sufficiently broad and durable systemic response.

This rationale underpins the combinations of RT with immune checkpoint inhibitors (ICIs). Preclinically, Dewan et al. showed that anti-CTLA-4 made abscopal responses more reproducible after fractionated RT, particularly with moderate hypofractionation (20). Mechanistically, Vanpouille-Box et al. later showed that regimens such as 8 Gy × 3 sustain cytosolic DNA sensing and IFN-I signaling, whereas very high single fractions may terminate this pathway through TREX1 induction (5). Thus, RT-ICI synergy depends on whether RT generates a priming-competent immune state and whether the host immune system can translate that state into effective T-cell expansion and trafficking.

Clinical evidence supports this concept but also highlights context dependence. In PEMBRO-RT, pembrolizumab preceded by SBRT to one lesion (8 Gy × 3) increased the 12-week response rate in non-irradiated lesions from 18% to 36% compared with pembrolizumab alone but the trial did not meet its prespecified threshold for meaningful clinical benefit and did not significantly improve survival in the overall cohort (39). PEMBRO-RT therefore supports proof of principle rather than broad clinical generalizability.

PACIFIC represents a distinct but highly influential setting: consolidation durvalumab after concurrent chemoradiotherapy for unresectable stage III NSCLC significantly improved progression-free survival, overall survival, and long-term outcomes, with 5-year overall survival of 42.9% versus 33.4% and progression-free survival of 33.1% versus 19.0% for durvalumab versus placebo (40–42). Although not a direct test of focal RT-induced abscopal biology, PACIFIC demonstrates that RT-based treatment can create a state in which PD-L1 blockade yields durable systemic benefit at scale.

Melanoma data further illustrate both promise and limitation. Postow et al. reported a landmark abscopal response after ipilimumab and palliative RT, but case reports cannot establish reproducibility (38). Prospective studies combining hypofractionated or dose-escalated RT with ipilimumab showed feasibility, systemic immune effects, and responses in subsets of patients, but overall activity remained modest (43, 44). Representative landmark trials evaluating the clinical synergy between RT and ICIs are summarized in **Table 4**.

**Table 4:** Landmark clinical RT-ICI studies.

Study/trial	Disease setting	RT approach	ICI	Main efficacy finding	Main interpretation/limitation
Postow et al., 2012 (38)	Metastatic melanoma case report	Local palliative RT	Ipilimumab	Classic clinical abscopal response with immune correlates	Proof of biologic plausibility, but anecdotal by design
PEMBRO-RT (Theelen et al., 2019) (39)	Advanced/metastatic NSCLC	SBRT to one lesion, 8 Gy × 3 before pembrolizumab	Pembrolizumab	12-week ORR 36% vs 18% with pembrolizumab alone	Supports synergy, but modest trial size and no definitive survival significance in overall cohort
PACIFIC (40–42)	Unresectable stage III NSCLC after concurrent CRT	Conventional thoracic CRT backbone	Durvalumab consolidation	Significantly prolonged OS, PFS after CRT	Landmark validation that RT-based therapy can synergize with PD-L1 blockade in curative-intent setting, though not a focal-abscopal design
Maity et al., 2021 (44)	Metastatic melanoma	Hypofractionated RT 6–8 Gy × 2–3 to one lesion	Ipilimumab	Feasible with systemic responses in subset	Supports combination feasibility and biologic activity, but modest efficacy and phase I scale
Boutros et al., 2020 (Mel-Ipi-Rx) (43)	Metastatic melanoma	Dose-escalated RT during ipilimumab	Ipilimumab	Feasible with systemic immune effects and non-irradiated lesion evaluation	Reinforces context-dependent synergy; not definitive for broad efficacy

Importantly, the landmark studies provide little direct evidence on how immunosenescence modifies RT–ICI efficacy or toxicity. PACIFIC reported exploratory subgroup analyses according to chronological age, but these analyses were not designed to assess biological immune aging. PEMBRO-RT and the smaller melanoma studies were not powered for robust comparisons between older and younger patients, and adults aged 75 years or older were underrepresented. Consequently, age-stratified data on response, survival, pneumonitis, lymphopenia, and other toxicities after combined RT–ICI remain limited. Meta-analytic evidence suggests that PD-1/PD-L1 inhibitors retain efficacy in conventionally defined older subgroups, generally aged 65 years or older, but these findings derive predominantly from ICI monotherapy or systemic-therapy trials and should not be extrapolated uncritically to RT–ICI combinations. Moreover, none of the landmark studies incorporated detailed baseline phenotyping of immunosenescence (45).

Overall, the abscopal effect is real but uncommon, and RT-ICI synergy is biologically and clinically plausible yet highly context-dependent. In older adults, its efficacy may additionally depend on biological immune age, preserved lymphocyte and DC function, comorbidity, and treatment tolerance. Effective radio-immunotherapy therefore requires treatment designs that preserve immune priming, limit lymphocyte depletion, counteract RT-induced suppressive feedback, and prospectively evaluate age-specific efficacy and toxicity (5, 22, 23, 39).

## Immunosenescence

### Hematopoiesis and innate myeloid cells

**Figure 2:**
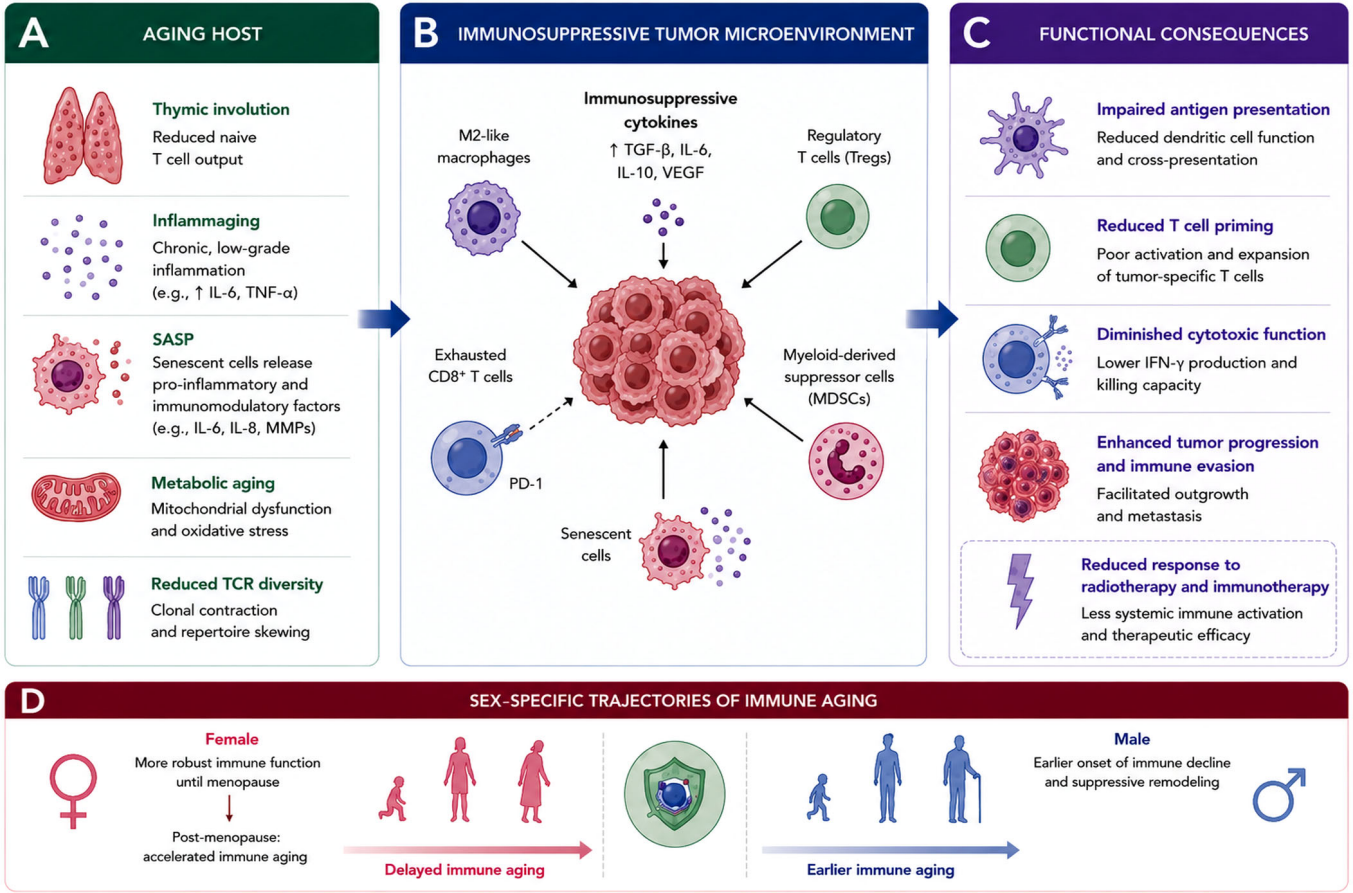
Biology of immunosenescence: age-associated remodeling of the immune system. **(A)** Aging-associated alterations in hematopoiesis promote myeloid-biased differentiation at the expense of lymphoid output, accompanied by increased DNA damage, replicative stress, and remodeling of the bone marrow microenvironment. These changes contribute to expansion of myeloid progenitors while reducing T-, B-, and natural killer (NK)-cell generation. **(B)** Innate immune aging is characterized by functional impairment of dendritic cells, macrophages, NK cells, and neutrophils, resulting in reduced antigen presentation, altered cytokine production, impaired cytotoxicity, and dysregulated inflammatory responses. **(C)** Adaptive immune aging involves thymic involution, contraction of the naïve T-cell compartment, reduced T-cell receptor (TCR) repertoire diversity, memory inflation, and accumulation of highly differentiated or senescent T cells. Aging-associated B-cell alterations include expansion of age-associated B cells (ABCs), impaired class-switch recombination, and reduced antibody responses. **(D)** Systemic features of immunosenescence include chronic low-grade inflammation (“inflammaging”), accumulation of senescent cells and senescence-associated secretory phenotype (SASP) factors, oxidative stress, mitochondrial dysfunction, and epigenetic alterations. Together, these interconnected processes drive progressive remodeling and functional decline of the aging immune system.

The immune system undergoes characteristic changes with age, which are collectively termed immunosenescence and impact every process from hematopoiesis to immune effector functions (46). The major biological hallmarks and cellular features of immunosenescence are summarized in **Figure 2**.

Immune cells differentiate from hematopoietic stem cells (HSCs) in the bone marrow. With increasing age, the bone marrow microenvironment changes, which is characterized by decreased cellular density, replacement of functional tissue by fat, and an altered cytokine milieu. Interestingly, the number of HSCs increases, but their capacity for self-renewal decreases (47), which can be partly explained by alterations in their energy metabolism. In addition, differentiation shifts towards the myeloid lineage, which gives rise to erythrocytes, thrombocytes, mast cells, granulocytes, monocytes/macrophages and dendritic cells, concomitant with a decline of common lymphoid progenitors (CLP), which differentiate into natural killer (NK) cells, T cells and B cells (48, 49). Aged HSCs accumulate DNA damage and mutations driven, e.g., by increased levels of reactive oxygen species, and display epigenetic changes. Changes in the bone marrow microenvironment, such as an altered cytokine milieu, further aggravate age-related dysregulation of hematopoiesis. As a consequence, the output of immune cells from the bone marrow, particularly of the lymphoid lineage, is decreased (50).

It was argued in the past that aging has only a minor effect on innate immune cells. A plethora of studies have shown that this is inaccurate and that functional defects in innate cells play an important role in the immunological dysregulation observed in old age. In the context of RT, innate immune cells serve as antigen-presenting cells and cytokine producers, promoting inflammation and the activation of adaptive immune cells. Functional defects in chemotaxis, alterations in the expression of pattern recognition receptors and their downstream signaling and altered cytokine production have been described for aged neutrophils (51), monocytes/macrophages (52), and dendritic cells (53). Antigen-presentation is diminished due to reduced expression of MHC class II. It has been shown that the composition of the circulating monocyte pool shifts towards intermediate and non-classical subsets. Intermediate monocytes have a high potential for tumor necrosis factor alpha (TNF-α) production, contributing to inflammaging (see below) (54). Circulating monocytes differentiate into macrophages when they exit the bloodstream and enter infected or inflamed tissue, whereas many tissue-resident macrophages localize to their tissue already during embryonic development (55). Macrophage characteristics are highly dependent on stimuli within their microenvironment and their activation state, and macrophages residing in different organs or tissues can have widely different phenotypes and functions (56). In combination with the difficulty of accessing human tissues, data on age-associated changes in specific tissue-resident cell populations are scarce and often inconsistent. A recent meta-analysis addressed these issues and aimed to identify common findings (57). Macrophages can – very simplified- be classified into pro-inflammatory M1 and anti-inflammatory M2 cells, particularly when studied *in vitro* (58), and a shift towards M1 phenotypes is observed with age. However, this only partially reflects the heterogeneity of this complex cell type *in vivo* and in different tissues. Most data on human innate immune cell function in old age have been generated using peripheral blood cells, which may not accurately reflect the tissue microenvironment. However, tissue-resident innate cells are of particular importance for tumor-specific immune responses.

### NK cells

NK cells play an important role in tumor surveillance and anti-tumor immune responses. They recognize altered cells, including virus-infected and tumor cells, using a range of activating and inhibitory receptors, and induce apoptosis in their target cells (59). Human NK cells can be classified as cytotoxic (CD56dim) and cytokine-producing (CD56bright) cells (60). With age, the number of NK cells decreases, and a shift towards more CD56dim and less CD56bright cells can be observed (61). Single-cell RNA expression analysis revealed a more complex picture (62–64) with more distinct subpopulations in blood and lymphoid organs and changes with age (65). As mentioned above, most studies on human immune cells utilize peripheral blood. A comprehensive study investigating NK cells across a wide range of human organs in autopsies describes tissue-specific NK cell properties but does not fully confirm the age-associated population shifts reported by many other studies (66). NK cells from older adults show decreased cytotoxic potential per cell (67) as well as reduced cytokine and chemokine production (68), which compromises their ability to eliminate tumor cells and to orchestrate responses of other immune cells. Aging of NK cells also leads to metabolic changes, which affect energy production and biosynthesis pathways and lead to altered mitochondrial function, increased ROS production, and cellular senescence (69). In addition to cell-intrinsic properties, the aged microenvironment, with decreased levels of IL-2 and IL-15 and increased levels of inflammatory cytokines such as IL-6, might further affect NK cell function.

### T cells

Age-related changes in the adaptive immune system are characterized by shifts in the composition of the T and B cell pools, with a transition from naïve to antigen-experienced cells. This is, per se, neither surprising nor detrimental, as we continuously encounter new antigens and build up a highly diverse memory repertoire over our lifetime, which is essential for rapid secondary responses. These changes are accompanied by a decrease in antigen-receptor diversity (repertoire). In addition, metabolic and signaling pathways are altered on a per-cell basis.

T cell precursors migrate from the bone marrow to the thymus, where somatic recombination of the T cell receptor (TCR) and positive and negative selection of functional T cells takes place. Mature naïve T cells leave the thymus and migrate to the periphery and secondary lymphoid organs (70). Thymic involution, the gradual replacement of functional thymic tissue by fat (71), starts early in life and leads to a dramatic decrease in the output of newly generated naïve T cells. Despite compensatory mechanisms, such as homeostatic proliferation, the frequency of naïve CD4+ and even more pronounced of CD8+ T cells is reduced in old age in the periphery and in lymphoid organs (32, 33). Even phenotypically naïve T cells from older adults show age-associated alterations, such as shortened telomeres (72) and reduced oxygen consumption rates (OCR), as well as attenuated glycolysis and mitochondrial metabolism upon activation (34). The number of different TCR sequences (specificities) in circulating cells is reduced in old age (73–75). This limited TCR repertoire hampers recognition of newly encountered antigens and recall responses (76). The frequency of antigen-experienced T cells increases with age, and, in particular, the accumulation of highly differentiated effector CD8+ T cells is a hallmark of immunological aging (77). Interestingly, the accumulation of CD4+ effector T cells is less pronounced and seems to occur later in life (78). Aging also alters the differentiation and distribution of CD4+ T cell subsets, including Th1, Th2, Th17, and Th9 cells with distinct distribution patterns observed in different tissues with aging (79, 80). Highly differentiated T cells display a more restricted cytokine profile, dominated by pro-inflammatory INF-γ and TNF-α (81), contributing to elevated inflammatory processes. In addition, DNA damage accumulates in aged T cells (82), highly differentiated T cells proliferate less in response to antigenic stimulation (30, 35) and display metabolic changes, such as decreased mitochondria, OCR, and mitochondrial membrane potential but increased mitochondrial and cytosolic ROS and anaerobic glycolysis (34). It has also been shown that cytotoxicity of CD8+ T cells decreases with age (83) due to a reduced frequency of cells that express perforin and granzyme B (84). In contrast, the number of cytotoxic CD4+ T cells increases with age (80).

As mentioned above, exhausted T cells, which are defined by the expression of PD-1, CTLA-4, and other co-inhibitory molecules, are highly relevant in the context of tumor-specific immune responses and immunotherapies. These cells arise under conditions of chronic stimulation with a single antigen, e.g., a tumor antigen, and are phenotypically and functionally distinct from highly differentiated or aged T cells, although there is some overlap, as these populations are all antigen-experienced.

Tregs play a critical role in the maintenance of peripheral tolerance and the prevention of autoimmunity but also diminish anti-tumor immune responses. The impact of age on Tregs is complex, as various distinct Treg subsets have been described in different steady state and pathological conditions (85).

### B cells

Aging is also associated with a shift in B cell subpopulations with a decrease in naïve B cells and an accumulation of antigen-experienced memory B cells and age-associated B cells (ABCs) (86). As mentioned above, increased numbers of antigen-experienced lymphocytes are beneficial for efficient secondary responses, but the overall decrease of repertoire diversity limits primary responses to neo-antigens (87). ABCs are defined by phenotypic markers such as CD11c expression and the transcription factor T-bet and have a distinct B cell receptor repertoire. They have been linked to enhancement of autoantibody production and autoimmune disease in older adults (88). In addition, this population produces pro-inflammatory cytokines such as IFN-γ and TNF-α, thereby contributing to inflammaging (89). It is not fully understood how ABCs arise. It has been suggested that they might be a memory subset induced by T-cell-dependent activation (90) or, alternatively, that they accumulate in an antigen-limited environment through homeostatic proliferation (91). Both hypotheses are based on murine data, and it should be taken into account that B-cell development and subsets differ substantially in humans. ABC-like B cells (CD27-IgD-IgG+ memory cells) accumulate in older adults (92). Whereas this study reported that the ABC-like B cell subset is not capable of antigen presentation, other studies conclude that ABCs are efficient antigen-presenting cells and that they may be responsible for increased Th17-polarization of T helper cells in older age (93, 94). B cells from older adults have decreased expression of E47 and activation-induced cytidine deaminase (AID), which are essential for class switch and somatic hypermutation in response to mitogenic stimulation, resulting in reduced IgG secretion (95).

Several crucial steps in B cell differentiation and antibody optimization, such as class switching, somatic hypermutation, and affinity maturation, as well as the generation of long-lived plasma cells, depend on T cell help in germinal centers. Impaired antibody responses in older adults are therefore caused not only by intrinsic changes in B cells but also by impaired T cell function.

### Inflammaging

At first sight contradictory to all the functional deficits of immune cells, aging is characterized by a chronic pro-inflammatory state (inflammaging), which contributes to the development and progression of many age-associated diseases, such as atherosclerosis, cardiovascular disease, cognitive decline, metabolic syndrome, and type 2 diabetes (96). However, impaired immunological function can lead to delayed clearance of pathogens and other target cells, resulting in prolonged activation and increased cytokine production. Alterations in the composition of innate immune cell subsets might favor pro- over anti-inflammatory populations and tissue damage, dysfunctional mitochondria, increased levels of reactive oxygen species, and accumulation of molecular damage to DNA and proteins with age lead to elevated inflammatory responses (97). Senescent cells accumulate with age due to increasing cellular damage, reduced repair mechanisms, and impaired clearance by immune cells (98). These cells secrete a plethora of soluble factors (SASP), including pro-inflammatory cytokines. Overall, baseline inflammatory levels in the absence of specific stimuli are higher in older adults (99).

## Conclusion

As the immune system is a complex network of various cells and soluble factors, it is important to keep in mind that functional alterations of a specific cell subset or dysregulation of a specific pathway inevitably impact many other components and functions. Immune cell distribution and phenotype are highly tissue-specific, and aging has differential impacts on the distinct local populations and their microenvironments (100). In addition, aging is not the only factor affecting the immune system. Underlying diseases, genetic variation, environmental factors, and the individual’s “immunbiography” - the sum of antigen contacts over time- shape the state of a person´s immune system. In the context of RT, the underlying tumor and anti-tumor therapies further impact immunological processes.

## How immunosenescence influences the anti-tumor immune response

**Figure 3:**
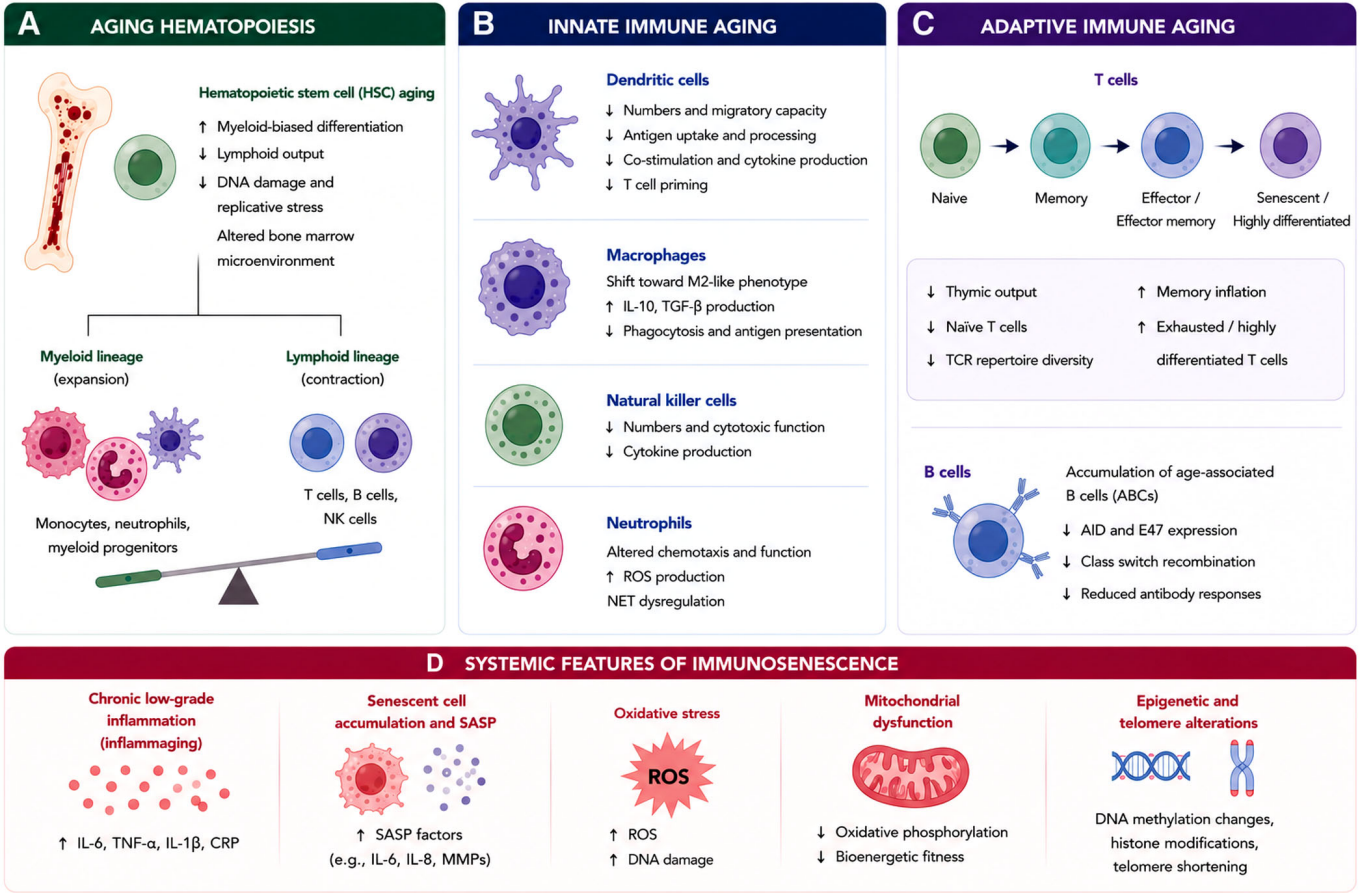
Immunosenescence-driven remodeling of anti-tumor immunity. **(A)** Hallmarks of the aging host contribute to the development of an immunosenescent state, including thymic involution with reduced naïve T-cell output, chronic low-grade inflammation (“inflammaging”), accumulation of senescent cells and senescence-associated secretory phenotype (SASP) factors, mitochondrial dysfunction and metabolic aging, as well as contraction of T-cell receptor (TCR) repertoire diversity. **(B)** In older individuals, the tumor microenvironment undergoes progressive immunosuppressive remodeling characterized by accumulation of M2-like macrophages, regulatory T cells (Tregs), myeloid-derived suppressor cells (MDSCs), senescent cells, and exhausted CD8+ T cells. This environment is further reinforced by immunosuppressive cytokines including TGF-β, IL-6, IL-10, and VEGF. **(C)** Functional consequences of immune aging include impaired antigen presentation and cross-priming, reduced activation and expansion of tumor-specific T cells, diminished cytotoxic effector function, enhanced tumor progression and immune evasion, and reduced responsiveness to radiotherapy and immunotherapy. **(D)** Immune aging follows sex-specific trajectories. Females generally maintain more robust immune function until menopause, followed by accelerated immune aging later in life, whereas males exhibit earlier onset of immune decline and suppressive immune remodeling. These sex-dependent differences may contribute to variability in anti-tumor immunity and therapeutic responsiveness in older patients.

Tumors develop within a dynamic tissue environment whose structural and immune signals can restrain or promote malignant behavior despite persistent oncogenic alterations (101–103). Aging remodels this environment through changes in immune-cell composition, inflammatory signaling, stromal function, and cellular senescence. In the context of RT, these alterations may impair the clearance of irradiated tumor cells, innate danger sensing, dendritic-cell activation, lymph-node priming, and T-cell effector responses. Immunosenescence may therefore reduce the probability that local radiation injury generates durable systemic immunity while favoring persistent inflammation and suppressive tissue remodeling. The principal mechanisms are summarized in **Figure 3**.

### Innate and myeloid immunosenescence in the irradiated tumor microenvironment

Although immunosenescence in oncology has traditionally focused on adaptive immunity and T-cell dysfunction (104), aging also alters macrophages, neutrophils, monocytes, and DCs that initiate and coordinate responses to radiation-induced tissue injury. These changes are heterogeneous and tissue-dependent, but may include impaired migration, phagocytosis, cytokine production, and inflammatory resolution, together with increased susceptibility to tumor-supportive polarization (51–53, 105).

Age-remodeled myeloid environments are relevant to cancers occurring predominantly in older adults, including non-small cell lung cancer (106), and can provide survival signals for malignant cells. In chronic lymphocytic leukemia, for example, nurse-like myeloid cells protect malignant B cells through chemokine and trophic signaling (105). In skeletal muscle, age-associated inflammation and impaired regenerative capacity may instead contribute to sarcopenia and cancer-associated cachexia, reducing physiological and immune reserve during treatment (107, 108). These examples illustrate the tissue-specific consequences of myeloid aging without implying a uniform macrophage phenotype across organs.

Myeloid dysfunction is particularly relevant after RT, because irradiated tumors release cellular debris, antigens, DAMPs, and nucleic acids that must be captured and processed by phagocytes and antigen-presenting cells. Impaired clearance may prolong inflammation without producing effective antigen presentation, while age-associated dendritic-cell dysfunction can weaken the transition from local injury to adaptive immunity. In aged tumor-bearing mice, DCs show impaired anti-tumor activity, whereas correction of age-associated defects can restore CD4+ T-cell-mediated tumor eradication (13, 14).

RT can itself reshape macrophage function in a context-dependent manner. Low-dose irradiation promoted an inflammatory, inducible nitric oxide synthase-expressing macrophage phenotype, vascular normalization, and T-cell recruitment in experimental tumors, whereas TH2-polarized CD4+ T cells and macrophages restricted RT efficacy in mammary carcinoma models. These findings indicate that radiation-induced myeloid remodeling may either support tumor rejection or reinforce tissue repair and immune suppression (109, 110).

Direct comparisons of myeloid responses to RT in young and aged tumor-bearing hosts remain limited. Consequently, whether aging preferentially promotes suppressive macrophage remodeling, defective dendritic-cell activation, or prolonged post-radiation inflammation remains incompletely defined.

### Adaptive immune aging: CD4+ help, CD8+ effector function, and regulatory T-cell balance

Parallel to innate immune remodeling, adaptive immunosenescence may directly restrict RT-induced anti-tumor immunity. Thymic involution reduces the generation of naïve T cells (111), while aging lymphoid organs acquire genomic, apoptotic, and epigenetic alterations that may impair immune-cell differentiation and plasticity (112). Older adults therefore increasingly rely on antigen-experienced peripheral T-cell populations, accompanied by contraction of the naïve pool and reduced T-cell receptor diversity (32, 33, 73–76). Because RT exposes tumor antigens that may require recognition by newly primed clones, these changes can reduce the probability that radiation-induced tumor injury generates a broad tumor-reactive response.

Age-associated CD4+ T-cell dysfunction is particularly relevant because effective anti-tumor priming depends on helper-cell-mediated dendritic-cell licensing, cytokine support for CD8+ T-cell expansion, and memory formation. Aging alters the differentiation and distribution of helper, regulatory, effector, and cytotoxic CD4+ subsets and can reduce their proliferative and metabolic responses after activation (30, 34, 35, 77). These changes may weaken lymph-node priming and reduce the durability of systemic immunity after RT (80, 113).

The CD8+ compartment is similarly affected. Naïve CD8+ T cells decline more markedly than naïve CD4+ cells, whereas highly differentiated populations accumulate and display reduced proliferative capacity, altered mitochondrial metabolism, increased DNA damage, and, in some studies, lower perforin and granzyme B expression (30, 32–35, 72, 82–84). These abnormalities may limit clonal expansion after RT-induced cross-priming and reduce cytotoxic control of irradiated and distant tumor sites.

Tregs further influence this balance. RT can enrich relatively radioresistant Tregs, while aging alters their abundance, phenotype, and suppressive function in a tissue- and context-dependent manner (26, 27, 85).

The relevant determinant may therefore be the post-RT balance between helper, cytotoxic, and Treg activity rather than Treg numbers alone.

Overall, adaptive immunosenescence can impair neoantigen recognition, CD4+ helper function, CD8+ expansion and cytotoxicity, and maintenance of tumor-reactive memory. The same radiation-induced antigenic stimulus may consequently produce a narrower or more regulatory-biased response in some older adults, although substantial interindividual heterogeneity remains.

### B cells, age-associated B cells, and tertiary lymphoid structures

Aging alters B-cell immunity through reduced naïve B-cell output, impaired class switching and somatic hypermutation, diminished antibody quality, and accumulation of ABCs or ABC-like populations (86–95). These changes may reduce repertoire diversity and antigen-presenting or humoral functions. However, the direct contribution of defective antibody responses to RT-induced anti-tumor immunity remains less established than dendritic-cell-mediated T-cell priming and should therefore not be considered a central mechanism of RT failure.

ABCs are heterogeneous populations associated with aging, chronic antigen exposure, inflammation, and autoimmunity. Although they can produce inflammatory cytokines and contribute to inflammaging, they should not be equated with all tumor-infiltrating B cells. B-cell function within tumors depends on phenotype, localization, antigen-presenting capacity, and interaction with T cells.

This distinction is important because tumor-infiltrating B cells and tertiary lymphoid structures (TLSs) have been associated with improved survival and responsiveness to immune checkpoint blockade in melanoma, sarcoma, and other tumors. These observations indicate that organized B-cell–T-cell niches can support productive local immunity rather than immune suppression (114–116).

The role of B cells and TLSs after RT remains insufficiently defined. RT may increase antigen availability and inflammatory recruitment, but there is no convincing evidence that it selectively recruits ABCs through bystander effects or that post-RT B-cell infiltration is uniformly suppressive. The immune consequence is more likely to depend on whether irradiation promotes coordinated antigen presentation and TLS-like organization or amplifies dysfunctional inflammatory B-cell programs in an aged microenvironment.

### Radiation-induced senescence in tumor, stromal, and immune compartments

Cellular senescence is a stress-response program characterized by durable proliferative arrest, persistent DNA-damage signaling, metabolic and chromatin remodeling, apoptosis resistance, and secretion of SASP mediators. Within the TME, senescent-marker-positive cells may include tumor cells, fibroblasts, endothelial cells, hematopoietic progenitors, myeloid cells, and lymphocytes. Accordingly, p16 ^INK4a^ or p16^High^ expression should be regarded as an important senescence-associated marker rather than as a cell-type-specific definition of senescence. p16^High^ senescence has been linked to restricted cellular plasticity in experimental reprogramming models (117), but senescence should ideally be defined using marker combinations and interpreted according to the affected cell population and functional phenotype (118).

Radiotherapy can induce senescence in tumor, stromal, endothelial, and hematopoietic compartments. In tumor cells, therapy-induced senescence (TIS) can initially support tumor control through durable growth arrest and, in some settings, enhanced immune surveillance. Immune clearance of senescent premalignant cells can restrict tumor development, illustrating the tumor-suppressive potential of senescence when surveillance is intact. However, persistent senescent tumor cells may secrete SASP factors that promote inflammation, matrix remodeling, stemness, and escape from growth arrest, thereby contributing to treatment resistance and relapse (118–120).

Normal tissue senescence provides a second, clinically important layer. Endothelial cells appear particularly relevant because they regulate vascular integrity, immune-cell trafficking, fibrosis, and tissue repair after RT. In human endothelial-cell models, irradiation induced a heterogeneous and dynamic senescence program that depended on dose and time, involved IL-1 signaling, and overlapped with endothelial-to-mesenchymal transition. In rats, unilateral renal irradiation induced glomerular senescence marked by SA-β-gal activity, p53/p21/p16 upregulation, loss of Ki-67, and an IL-6-dominated SASP, with glomerular endothelial injury, thrombotic microangiopathy, collapsing glomeruli, and chronic renal dysfunction. These data support the view that RT-induced endothelial senescence can contribute to organ injury and may indirectly shape the immune context by promoting vascular dysfunction and chronic inflammatory signaling (121, 122).

Radiation-induced senescence may also promote fibrosis through paracrine signaling. In a p16-reporter model, radiation-induced senescent tdTOM-p16-positive mesenchymal cells acquired a distinct transcriptional profile and induced profibrotic genes in non-contact co-cultures. Fgr, a member of the Src family kinases, was markedly upregulated in senescent cells. Fgr inhibition did not prevent senescence itself but blocked senescent-cell induction of profibrotic mediators such as TGF-β and collagen. *In vivo*, single-cell RNA sequencing after thoracic irradiation identified Fgr expression in senescent myeloid populations, including neutrophils and macrophages, before the development of radiation-induced pulmonary fibrosis. This finding is relevant to immunosenescence because it links radiation-induced senescent stromal or myeloid programs to chronic tissue remodeling rather than acute immune activation (123).

Senescence may also affect hematopoietic and immune compartments after genotoxic stress. Ionizing radiation can induce long-term dysfunction and senescence-like features in hematopoietic stem and progenitor cells, potentially reducing the regenerative capacity needed to restore lymphocyte pools after treatment. In immune cells, senescence or terminal differentiation may impair proliferation, alter cytokine production, and weaken coordinated anti-tumor function. These effects may complement radiation-induced lymphopenia: lymphopenia reduces immune-cell numbers, whereas senescence diminishes the quality and regenerative potential of the surviving compartment (124, 125).

The SASP links these compartments and can exert opposing effects. Acute SASP signaling may recruit immune cells, facilitate clearance of damaged cells, and support tissue repair. In contrast, persistent SASP signaling can sustain IL-6-, IL-1-, TGF-β-, chemokine-, and matrix-remodeling programs that promote suppressive myeloid recruitment, fibrosis, vascular dysfunction, and impaired effector-cell function. In older adults, RT may therefore add to an already senescence-rich and inflammatory tissue environment, shifting the response from transient immune activation toward chronic immune dysregulation and tissue injury (126).

This dual role argues against indiscriminate senescence suppression. Beneficial acute growth arrest, immune surveillance, and tissue repair should be distinguished from persistent senescent-cell burden and maladaptive SASP signaling. Potential senolytic, senomorphic, and targeted-clearance strategies and should therefore be considered in a temporal- and compartment-specific manner and are discussed in the therapeutic section.

### Systemic metabolic aging, inflammaging, and geroncogenesis

RT-induced anti-tumor immunity requires metabolic adaptation by DCs and tumor-reactive lymphocytes. Aging is associated with impaired mitochondrial activation, reduced glycolytic and oxidative responses, and chronic inflammaging, which may limit T-cell clonal expansion and effector function after RT-induced priming (34, 96–99).

Broader age-related changes in systemic metabolism may also promote tumor progression, a concept termed geroncogenesis (127). In aged mice, declining nicotinamide adenine dinucleotide (NAD+) disrupted nuclear–mitochondrial communication through a SIRT1-dependent mechanism and contributed to impaired mitochondrial homeostasis. Although this provides a mechanistic link between NAD+ decline, sirtuin activity, and metabolic aging, direct evidence that this pathway determines immune responses to RT is currently lacking (128).

Age-associated circulating metabolites may additionally act directly on malignant cells. Gomes et al. showed that methylmalonic acid accumulates in the circulation with age and can induce SOX4-dependent transcriptional reprogramming, epithelial–mesenchymal transition-like features, and increased tumor aggressiveness (129). This finding illustrates how the aged systemic environment may promote tumor progression independently of local immune-cell dysfunction. It does not, however, establish that methylmalonic acid induces TGF-β secretion or directly suppresses RT-induced immunity.

Systemic metabolic aging may therefore influence responses to RT by reducing immune-cell bioenergetic competence, sustaining chronic inflammatory signaling, and promoting tumor-cell programs that resist immune clearance. Most of these mechanisms have not been examined directly in irradiated older hosts and should be considered plausible modifiers rather than established predictors of RT or RT–ICI response.

### Sexual dimorphism and hormonal modifiers of RT-induced anti-tumor immunity

Biological sex influences innate and adaptive immunity through sex chromosomes, gonadal hormones, and age-related endocrine changes (130–134). The X chromosome contains several immune-regulatory genes, including TLR7 and FOXP3, and incomplete X-chromosome inactivation may increase expression of selected genes in female immune cells. For example, TLR7 can escape X-chromosome inactivation and enhance innate immune responses in subsets of female cells. These differences may affect inflammatory signaling and antigen presentation, but do not imply uniformly superior anti-tumor immunity in either sex (135).

Estrogens can enhance selected dendritic-cell, lymphocyte, and interferon responses, but may also promote regulatory or anti-inflammatory effects depending on concentration, receptor expression, and tissue context (131–133). Menopause alters this hormonal and inflammatory environment, while age-associated contraction of naïve lymphocytes and accumulation of ABCs further remodel immunity (134, 136). Conversely, androgen signaling can favor suppressive immune programs involving Tregs, MDSCs, macrophages, and dysfunctional CD8+ T cells in selected tumor contexts (131–133, 137). T-cell-intrinsic androgen-receptor signaling has been shown to reinforce CD8+ T-cell exhaustion and contribute to sex differences in tumor progression and checkpoint-inhibitor response (138).

These mechanisms may be relevant to RT because radiation-induced immunity depends on innate nucleic-acid sensing, interferon production, dendritic-cell activation, T-cell priming, and inflammatory resolution. Sex-related differences could therefore modify the magnitude of immune activation, macrophage and Treg responses, fibrosis, or susceptibility to treatment-related inflammation. However, direct RT-specific evidence remains limited, and most experimental and clinical studies have not separated chromosomal, hormonal, age-related, and tumor-specific effects. Biological sex should therefore be regarded as a potential modifier rather than a deterministic predictor of RT or RT–ICI response.

Taken together, immunosenescence may shift the response to RT from coordinated anti-tumor immune activation toward incomplete priming, reduced effector function, and persistent inflammatory or suppressive remodeling. Impaired antigen presentation, restricted T-cell receptor diversity, reduced CD4+ help, diminished CD8+ proliferative capacity, Treg and myeloid suppression, chronic SASP signaling, metabolic dysfunction, and sex-dependent immune differences may each contribute to this altered balance. However, these mechanisms vary substantially between individuals and cannot be inferred from chronological age alone. Direct human evidence linking immune-aging phenotypes to RT-induced immune responses or RT–ICI outcomes remain limited; most available data derive from aging studies without RT, RT-immunology studies in young experimental models, or cellular senescence models. Immune age should therefore be considered an emerging biomarker concept and hypothesis-generating framework rather than an established clinical stratifier.

### Therapeutic strategies to enhance radiation-induced anti-tumor immunity in immunosenescent patients

The ability of RT to induce systemic anti-tumor immune responses depends on efficient activation of the cancer immunity cycle, including antigen release, dendritic cell priming, and expansion of tumor-specific cytotoxic T cells. As discussed in the previous sections, aging-associated alterations of the immune system can modulate several of these processes and may thereby influence the magnitude and durability of radiation-induced anti-tumor immunity. In particular, age-related changes in T-cell composition, antigen-presenting cell function, and TME architecture may limit the capacity of RT to generate effective systemic immune responses, including abscopal effects, and may alter the efficacy of combination strategies with ICIs. Current clinical decision-making in both medical and radiation oncology largely relies on chronological age and general biological fitness, whereas immunological age is rarely considered despite its potential relevance for treatment response and immune competence.

**Figure 4:**
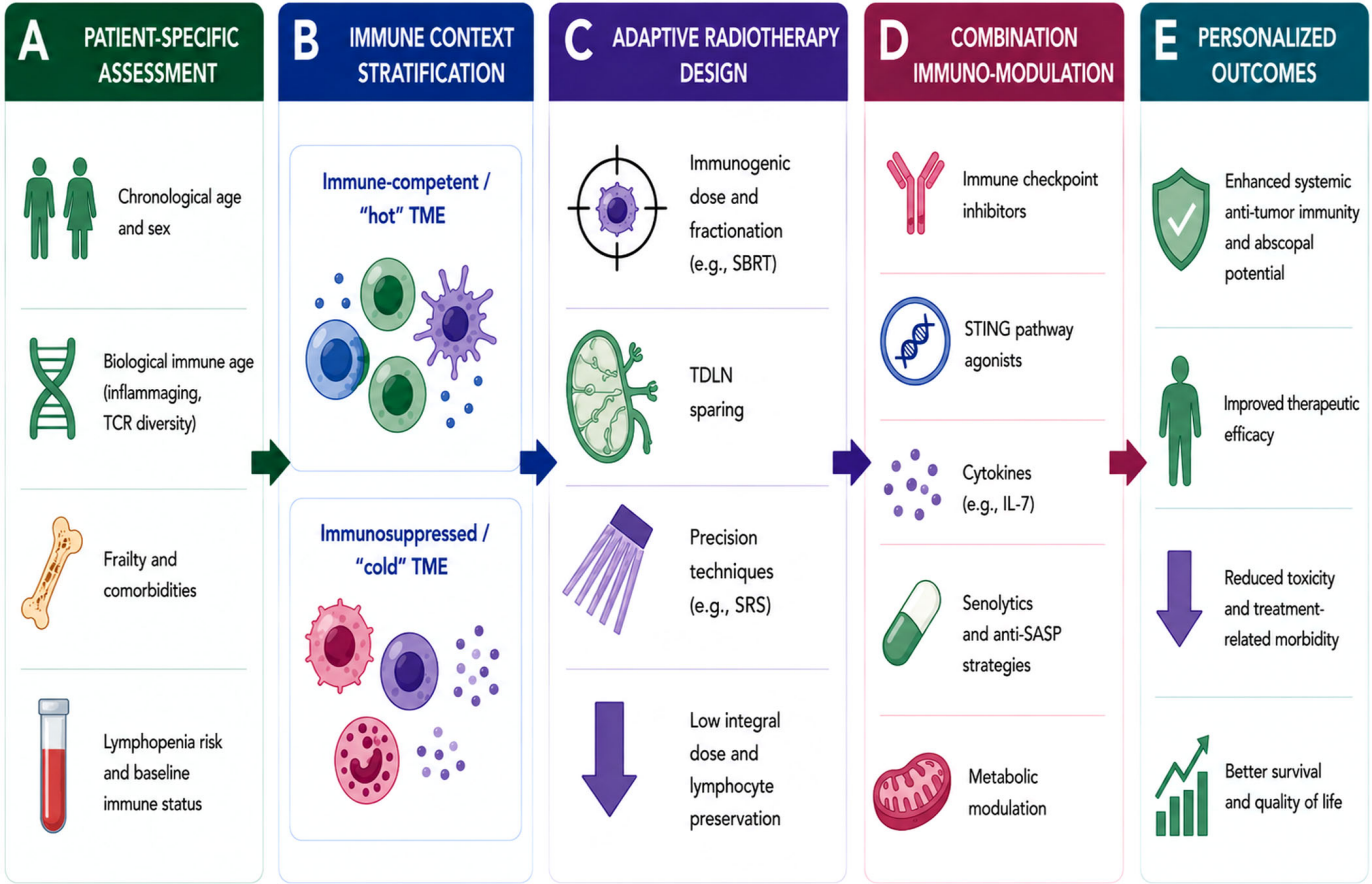
Personalized radio-immunotherapy strategies in the aging host. **(A)** Patient-specific assessment includes evaluation of chronological age, sex, biological immune age, frailty, comorbidities, lymphopenia risk, and baseline immune status. Biological immune age may be reflected by features such as inflammaging and reduced T-cell receptor (TCR) diversity. **(B)** Immune context stratification distinguishes between immune-competent (“hot”) and immunosuppressed (“cold”) tumor microenvironment (TME), enabling characterization of the baseline immune landscape prior to therapy selection. **(C)** Adaptive radiotherapy design aims to optimize immune preservation and immunogenicity through individualized treatment strategies, including immunogenic dose and fractionation schedules (e.g., stereotactic body radiotherapy, SBRT), tumor-draining lymph node (TDLN) sparing, precision techniques (e.g., stereotactic radiosurgery, SRS), and reduction of integral dose to preserve circulating lymphocytes. **(D)** Combination immuno-modulatory approaches may enhance therapeutic efficacy through integration of immune checkpoint inhibitors, STING pathway agonists, cytokine-based therapies such as IL-7, senolytic or anti-senescence-associated secretory phenotype (SASP) strategies, and metabolic modulation. **(E)** Personalized treatment approaches may improve systemic anti-tumor immunity and abscopal potential, increase therapeutic efficacy, reduce treatment-related toxicity and morbidity, and ultimately improve survival and quality of life in older cancer patients.

These observations highlight the need for therapeutic approaches that address age-related constraints of anti-tumor immunity in the context of RT. Because immunosenescence comprises heterogeneous and potentially targetable biological processes, several strategies are currently being explored to enhance radiation-induced immune activation in older patients, including optimization of RT parameters to preserve immune competence, rational combination with immunotherapies, and interventions targeting hallmarks of immune aging such as chronic inflammation, cellular senescence, and metabolic dysregulation. Emerging evidence suggests that modulation of these pathways may improve treatment responsiveness and support the development of biomarker-guided personalized radio-immunotherapy approaches for older patients (139, 140). A conceptual framework for personalized radio-immunotherapy in the aging host is summarized in **Figure 4**.

In this section, we discuss current and emerging therapeutic strategies that may enhance radiation-induced anti-tumor immune responses in immunosenescent patients, with particular emphasis on RT optimization, combination immunotherapies, and biomarker-driven personalization concepts.

### Optimizing radiotherapy to preserve immune competence and enhance radiation-induced anti-tumor immunity

One of the most direct strategies to enhance radiation-induced anti-tumor immunity in immunosenescent patients is the preservation of functional immune cell populations during RT. While RT can stimulate anti-tumor immune responses through immunogenic cell death, antigen release, and activation of dendritic cells, it may simultaneously induce systemic immunosuppression, most prominently in the form of radiation-induced lymphopenia (RIL) (141). Given that lymphocytes are among the most radiosensitive cell types in the human body, even relatively low radiation doses can substantially reduce circulating T-cell pools, thereby potentially limiting the ability of patients to mount effective anti-tumor immune responses (142).

Accumulating clinical evidence suggests that treatment-associated lymphopenia correlates with inferior oncologic outcomes across multiple tumor entities, including glioblastoma, esophageal cancer, head and neck squamous cell carcinoma (HNSCC), brain metastases and cervical cancer (143–148). Reduced lymphocyte counts during RT have been associated with impaired tumor control, decreased overall survival, and potentially reduced responsiveness to ICIs. These findings highlight the importance of preserving immune competence as a prerequisite for effective radio-immunotherapy combinations (149–151).

Mechanistically, RT-induced lymphopenia may result not only from direct irradiation of circulating lymphocytes but also from exposure of lymphoid organs such as draining lymph nodes, spleen, thymic remnants, and bone marrow. Furthermore, large irradiation volumes and prolonged fractionation schedules may increase the probability that circulating immune cells repeatedly pass through irradiated fields, thereby amplifying systemic immune depletion. This has led to the conceptual expansion of classical organs-at-risk (OAR) to include structures relevant for immune function, sometimes referred to as immune organs-at-risk (iOAR) (152, 153). Beyond radiation-induced lymphopenia, RT can also induce cellular senescence in multiple non-malignant cellular compartments, including endothelial, stromal, and immune cells (121, 123). Radiation-induced senescence is increasingly recognized as a double-edged process: while stable growth arrest may contribute to tumor suppression, the accumulation of senescent cells can promote the release of SASP factors, including IL-1-, IL-6-, and other inflammatory mediators (121, 122). Persistent SASP signaling may contribute to chronic inflammation, tissue injury, fibrosis, and immunosuppressive remodeling of the TME (122, 123). Importantly, these effects may be particularly relevant in older patients, who already exhibit increased senescent-cell burden and inflammaging. Therefore, radiation-induced senescence represents an additional mechanism by which RT may influence immune competence beyond direct lymphocyte depletion and may provide a rationale for future combinations with senolytic or anti-SASP interventions.

Dosimetric and technical parameters of RT appear to play an important role in determining the degree of immune suppression. Larger treatment volumes, extended-field irradiation, and increased integral dose have been associated with more pronounced lymphopenia. For example, more extensive radiation fields have been linked to stronger reductions in circulating lymphocyte counts and worse survival outcomes in patients with various solid cancers (154). Similarly, dose-distribution parameters such as low-dose exposure to large tissue volumes (e.g., V5Gy) may contribute substantially to depletion of immune effector cells (155).

Consequently, several RT optimization strategies are currently being explored to preserve immune competence, including reduction of irradiated volumes, selective sparing of lymphoid structures, hypofractionated treatment regimens, stereotactic approaches, and proton therapy to reduce integral dose (156–158) Importantly, the potential immunological advantages associated with proton therapy are primarily attributable to improved sparing of healthy lung tissue, particularly reduced low-dose exposure (e.g., lower lung V5Gy), rather than to unique biological properties of proton radiation itself. Instead, these effects are likely driven by the superior dose conformity and steep dose gradients achievable with highly precise treatment techniques. Consequently, other advanced high-precision RT modalities, such as stereotactic body radiotherapy or stereotactic radiosurgery, may confer similar immunological benefits through comparable reduction of normal tissue and circulating immune cell exposure. In addition, advanced image-guided and adaptive RT techniques may allow more precise targeting of tumor tissue while minimizing exposure of circulating immune cells. Collectively, these approaches aim to maintain sufficient immune functionality to support radiation-induced priming of anti-tumor T-cell responses, which may be particularly relevant in older patients with already limited immune reserve.

Preservation of immune competence during RT may therefore represent a critical prerequisite for maximizing synergy between RT and immunotherapy and could contribute to improved personalization of treatment strategies in older cancer patients. Recent reviews further emphasize that radiation-induced immune modulation comprises both immunostimulatory and immunosuppressive effects, highlighting the importance of treatment strategies that preserve beneficial anti-tumor immune responses while minimizing treatment-related immune dysfunction (159). The relative importance of immune-preserving RT strategies may be particularly pronounced in older patients, who frequently exhibit reduced immune reserve due to immunosenescence, diminished naïve T-cell pools, and age-associated alterations in adaptive immunity (32, 33, 73–76). In this setting, prevention of radiation-induced lymphopenia and preservation of lymphoid structures may become especially relevant, as recovery of adaptive immune function may be less robust than in younger individuals. The optimal prioritization of these approaches is likely to depend on tumor location and treatment volume. For example, thoracic irradiation is frequently associated with substantial exposure of circulating blood volume and pulmonary immune compartments, whereas central nervous system irradiation may involve distinct patterns of immune and lymphoid tissue exposure. Although direct comparative clinical evidence remains limited, these considerations support the development of site-specific and age-adapted RT strategies aimed at maximizing immune preservation while maintaining oncologic efficacy (152–155).

### Immune checkpoint blockade to reinforce radiation-induced anti-tumor immunity in immunosenescent patients

ICIs represent a central strategy to enhance radiation-induced anti-tumor immunity by restoring T-cell effector function and overcoming inhibitory signaling pathways within the TME. As discussed in previous sections, aging is associated with the accumulation of antigen-experienced and highly differentiated T-cell populations that frequently exhibit increased expression of inhibitory receptors such as PD-1, CTLA-4, and TIM-3 (160, 161). Although these phenotypic changes are often interpreted as a sign of reduced immune competence, they may also create a therapeutically exploitable vulnerability, as checkpoint inhibition can partially reinvigorate pre-existing tumor-reactive T cells even in aged immune systems (162). As described above, RT may further enhance responsiveness to ICI by increasing tumor immunogenicity through induction of immunogenic cell death, release of tumor-associated antigens, and activation of innate immune sensing pathways. Preclinical evidence suggests that RT promotes dendritic cell activation and T-cell priming, thereby converting poorly infiltrated (“cold”) tumors into more inflamed (“hot”) TMEs that may be more susceptible to immune checkpoint inhibition. In addition, RT has been shown to increase PD-L1 expression in tumor and immune cells, providing a mechanistic rationale for combined radio-immunotherapy approaches.

Clinical evidence indicates that older patients can benefit from immune checkpoint inhibitors, although outcomes appear heterogeneous depending on tumor type, treatment combination, and patient fitness. Several analyses suggest comparable efficacy of PD-1/PD-L1 inhibitors in patients aged ≥65 years relative to younger cohorts, particularly when administered as monotherapy (45). However, evidence in patients ≥75 years remains limited, as older individuals are frequently underrepresented in clinical trials (163). Similarly, meta-analytic data indicate that addition of ICI to systemic therapy improves overall survival in selected tumor types in older patients, although benefits vary across cancer entities and patient populations (164). The combination of RT and ICI is currently being extensively investigated in prospective clinical trials, with the goal of exploiting synergistic interactions between local tumor irradiation and systemic immune activation. Analyses of combined RT and checkpoint blockade therapies suggest manageable toxicity profiles, although treatment-related adverse events such as pneumonitis, fatigue, and lymphopenia require careful monitoring, particularly in older patients with limited physiological reserve (45, 165). Importantly, management of treatment-related adverse events represents a particular challenge in older patients receiving combined radiotherapy and immune checkpoint inhibitors. Immune-related adverse events are commonly treated with systemic corticosteroids or other immunosuppressive agents, which may theoretically compromise anti-tumor immunity by attenuating T-cell activation and effector function and have been associated with reduced efficacy of immune checkpoint inhibitors in some clinical settings (166, 167). Likewise, treatment interruptions or dose modifications implemented to reduce toxicity may diminish the immunostimulatory effects intended by radio-immunotherapy. Balancing effective toxicity management with preservation of anti-tumor immune responses therefore represents an important clinical consideration, particularly in older patients with limited physiological reserve and pre-existing organ dysfunction. Future studies are needed to better define strategies that mitigate treatment-related toxicity while maintaining therapeutic immune activation.

While reduced naïve T-cell output may limit *de novo* priming of anti-tumor responses, the presence of antigen-experienced T cells with increased expression of inhibitory receptors suggests that checkpoint blockade may remain effective in selected older patients. Consequently, rational integration of ICI into radio-immunotherapy regimens may help compensate for age-associated limitations in endogenous anti-tumor immunity and support the development of personalized treatment strategies based on immune competence rather than chronological age alone.

Future research is needed to better define predictive biomarkers of response to ICI in older patients, including immune repertoire diversity, inflammatory signatures, and expression patterns of inhibitory receptors within the TME.

### Targeting hallmarks of immunosenescence to enhance radiation-induced anti-tumor immunity

While optimization of RT delivery and the integration of immune checkpoint blockade represent important strategies to enhance anti-tumor immunity in older patients, immunosenescence affects multiple cellular and molecular pathways that extend beyond T-cell inhibitory receptor signaling. As described above, age-associated alterations in HSC differentiation, thymic involution, chronic low-grade inflammation (“inflammaging”), and accumulation of senescent immune cells collectively reshape the TME and may limit the efficacy of radiation-induced anti-tumor immune responses. Consequently, therapeutic strategies targeting fundamental hallmarks of immunosenescence may complement radio-immunotherapy approaches and improve clinical outcomes in older cancer patients.

A central feature of immunosenescence is the progressive decline in naïve T-cell output due to thymic involution, resulting in restricted T-cell receptor repertoire diversity and impaired priming of tumor-specific immune responses. This limitation may be particularly relevant in the context of RT, which relies on efficient antigen presentation and priming of novel tumor-specific T-cell clones following immunogenic cell death. Strategies aimed at restoring thymopoiesis or enhancing T-cell regeneration, such as interleukin-7 (IL-7)-based approaches, have therefore been proposed to improve immune competence in older individuals. Preclinical studies suggest that restoration of thymic function or expansion of stem-like T-cell populations may enhance anti-tumor immunity and improve responsiveness to immunotherapy (168, 169).

Another hallmark of immunosenescence is chronic systemic inflammation, often referred to as inflammaging, characterized by persistent activation of NF-κB signaling pathways and increased production of pro-inflammatory cytokines such as IL-6 and TNF-α. This inflammatory milieu promotes accumulation of immunosuppressive cell populations, including MDSCs and Tregs, which can impair radiation-induced T-cell priming and effector function. Targeting inflammatory signaling pathways through metabolic or pharmacologic interventions, including mTOR inhibition or modulation of cellular metabolism, has been proposed as a strategy to restore immune homeostasis and improve anti-tumor immune responses in older individuals (170–172).

Accumulation of senescent cells and the development of a SASP further contribute to age-related immune dysfunction and tumor progression. SASP-associated cytokines, chemokines, and growth factors can promote tumor growth, impair antigen presentation, and foster an immunosuppressive TME. Emerging therapeutic strategies targeting senescent cells, including senolytic agents or senomorphic drugs that modulate SASP signaling, have shown promise in preclinical models and may enhance responsiveness to RT and immunotherapy combinations (173–177).

In addition to adaptive immune dysfunction, age-related alterations in innate immune cell populations contribute to impaired anti-tumor immunity. Dendritic cells in older individuals exhibit reduced antigen presentation capacity and impaired type I interferon responses, potentially limiting efficient priming of tumor-specific T cells following radiation-induced immunogenic cell death. Therapeutic activation of innate immune sensing pathways, including stimulation of Toll-like receptors or the cGAS-STING pathway, has been proposed to enhance dendritic cell activation and promote anti-tumor T-cell responses in older hosts. These strategies may be particularly relevant in the context of RT, which induces cytosolic DNA accumulation and activation of innate immune signaling pathways (1, 140, 178).

Collectively, therapeutic targeting of immunosenescence-associated pathways may enhance the immunogenic effects of RT by improving antigen presentation, restoring T-cell function, and reducing immunosuppressive signaling within the TME. Importantly, interventions targeting biological aging processes may complement immune checkpoint blockade strategies and provide synergistic opportunities to enhance treatment efficacy in older cancer patients.

Future research should aim to identify biomarkers reflecting biological immune age, including immune repertoire diversity, inflammatory signatures, and senescence-associated transcriptional programs, to guide personalized selection of immunomodulatory interventions in combination with RT.

### Biomarkers of immune aging to guide personalized radio-immunotherapy

Chronological age alone is an insufficient predictor of immune competence and treatment response in cancer patients. Substantial interindividual variability exists in the degree and phenotype of immunosenescence, resulting in heterogeneous responses to RT, immune checkpoint blockade, and combination strategies. Consequently, identification of biomarkers reflecting biological immune age may enable improved stratification of patients and facilitate personalized radio-immunotherapy approaches. Recent evidence suggests that integration of immune profiling into clinical decision-making may help distinguish patients with preserved immune responsiveness from those who may benefit from targeted interventions addressing immunosenescence-associated dysfunction (139, 179, 180).

Several candidate biomarkers reflecting adaptive immune aging have been proposed, including alterations in T-cell subset composition, reduced TCR repertoire diversity, and increased frequencies of highly differentiated CD8+CD28- T cells (181). Age-associated shifts in the CD4/CD8 ratio and contraction of the naïve T-cell pool may indicate impaired capacity to mount *de novo* immune responses following radiation-induced antigen release. These alterations may be particularly relevant for predicting responsiveness to therapies relying on efficient priming of tumor-specific T cells (182).

Inflammaging-related biomarkers, including elevated circulating levels of IL-6, TNF-α, and C-reactive protein (CRP), have been associated with frailty and age-related immune dysregulation. Chronic systemic inflammation may promote expansion of immunosuppressive cell populations such as MDSCs and Tregs, thereby impairing radiation-induced anti-tumor immune responses. Systematic analyses indicate that IL-6 and CRP are among the most consistently observed biomarkers associated with immune aging and functional decline in older individuals (183).

Emerging multi-omics approaches provide additional opportunities to characterize immune aging through integrated transcriptomic, epigenetic, and proteomic analyses. Single-cell RNA sequencing studies have identified senescence-associated gene expression signatures, including increased expression of cell cycle inhibitors such as p16INK4a and p21, as well as altered cytokine signaling pathways associated with inflammaging. These molecular signatures may enable improved characterization of immune system heterogeneity in older patients and facilitate identification of therapeutic vulnerabilities (184–186). Rather than relying on individual biomarkers, future approaches will likely require integration of multiple dimensions of immune aging into a composite assessment of biological immune age. Such a framework could combine immune parameters (e.g., T-cell subset composition and TCR repertoire diversity), inflammatory markers (e.g., IL-6, TNF-α, and CRP), metabolic features associated with immunosenescence, and molecular signatures derived from transcriptomic or epigenetic profiling. Although no standardized immune-age classification currently exists, multidimensional biomarker models may allow more accurate identification of patients with preserved immune competence versus those with advanced immunosenescence, thereby supporting personalized radio-immunotherapy strategies.

Integration of immune aging biomarkers into clinical decision frameworks may support individualized treatment strategies in the context of RT. For example, patients with preserved TCR diversity and limited inflammatory signatures may benefit from standard radio-immunotherapy approaches, whereas patients with pronounced inflammaging or reduced naïve T-cell pools may require additional immunomodulatory interventions, such as cytokine therapies, metabolic modulation, or strategies targeting myeloid-derived immunosuppression. Future clinical trials should therefore incorporate immune profiling to determine whether biomarker-guided personalization of radio-immunotherapy improves outcomes in older cancer patients.

Biomarkers reflecting biological immune age may improve patient stratification and guide personalized radio-immunotherapy approaches beyond chronological age alone. Integration of immunological, inflammatory, and molecular biomarkers into clinical decision-making frameworks may help identify older patients most likely to benefit from specific therapeutic strategies. Future studies incorporating immune profiling into clinical trials will be essential to enable precision immuno-oncology approaches tailored to aging immune systems. Collectively, biomarkers reflecting biological immune age may support patient stratification and facilitate personalized radio-immunotherapy approaches beyond chronological age alone.

### Future perspectives: integrating immunosenescence into personalized radio-immunotherapy

The growing recognition that immunosenescence significantly shapes anti-tumor immune responses highlights the need for more precise integration of aging biology into radio-immunotherapy strategies. Current treatment paradigms incompletely capture the heterogeneity of immune competence in older patients with cancer. Future approaches will likely require multidimensional patient stratification incorporating immunological biomarkers, clinical parameters, and molecular profiling to guide individualized therapeutic decisions.

Prospective clinical trials specifically designed for older patients remain limited, and older individuals are frequently underrepresented in studies evaluating combined RT and immunotherapy approaches. Improved trial design incorporating biological age metrics, immune profiling, and age-adapted endpoints may help identify patient subgroups most likely to benefit from combined modality treatment. In particular, integration of longitudinal immune monitoring may provide insights into dynamic changes in immune competence during RT and help optimize treatment sequencing and dosing. Future clinical trials should move beyond chronological age alone and incorporate prospective immune phenotyping as part of study design. Baseline assessment of immune competence, including T-cell composition, TCR repertoire diversity, inflammatory biomarkers, and other indicators of biological immune age, may help identify patient populations most likely to benefit from radio-immunotherapy. In addition, immune-aging biomarkers could be incorporated as exploratory endpoints or stratification factors to better understand treatment heterogeneity among older patients. Dedicated trials specifically designed for older adults will be essential to determine whether biomarker-guided treatment adaptation can improve both efficacy and tolerability.

Emerging technologies, including single-cell transcriptomics, spatial immunoprofiling, and systems biology approaches, offer new opportunities to better characterize the aging tumor immune microenvironment and identify actionable therapeutic targets. Such approaches may help distinguish adaptive versus maladaptive features of immune aging and enable rational design of combination therapies tailored to individual immune phenotypes.

Importantly, aging is frequently accompanied by comorbidities, polypharmacy, and altered tissue repair capacity, all of which may influence tolerance to RT and immunotherapy. Therefore, interdisciplinary collaboration between radiation oncologists, immunologists, and geriatric specialists will likely be essential for developing clinically applicable personalized immuno-oncology strategies for older patients.

Collectively, integration of immunosenescence into treatment decision-making represents a key step toward biologically informed precision oncology and may improve outcomes for the growing population of older cancer patients receiving RT and/or immunotherapy.
